# Maximal oxygen uptake prediction from submaximal bicycle ergometry using a differential model

**DOI:** 10.1038/s41598-023-38089-7

**Published:** 2023-07-12

**Authors:** Monika Petelczyc, Michał Kotlewski, Sven Bruhn, Matthias Weippert

**Affiliations:** 1 Faculty of Physics, Warsaw University of Technology, Koszykowa 75, 00-662 Warsaw, Poland; 2 Institute of Sport Science, University of Rostock, Ulmenstrasse 69, Rostock, Germany

**Keywords:** Cardiovascular biology, Scientific data, Biological physics

## Abstract

The maximal oxygen uptake (VO_2_max) estimation has been a subject of research for many years. Cardiorespiratory measurements during incremental tests until exhaustion are considered the golden yard stick to assess VO_2_max. However, precise VO_2_max determination based on submaximal tests is attractive for athlete as well for clinical populations. Here, we propose and verify such a method based on experimental data. Using a recently developed model of heart rate (HR) and VO_2_ kinetics in graded exercise tests, we applied a protocol, which is terminated at 80% of the estimated maximal HR during ergometer cycling. In our approach, initially, formula for maximal HR is selected by retrospective study of a reference population (17 males, 23.5 ± 2.0 years, BMI: 23.9 ± 3.2 kg/m^2^). Next, the subjects for experimental group were invited (nine subjects of both sexes: 25.1 ± 2.1 years, BMI 23.2 ± 2.2 kg/m^2^). After calculation of maximal HR using cardiorespiratory recordings from the submaximal test, VO_2_max is predicted. Finally, we compared the prediction with the values from the maximal exercise test. The differences were quantified by relative errors, which vary from 1.2% up to 13.4%. Some future improvements for the procedure of VO_2_max prediction are discussed. The experimental protocol may be useful for application in rehabilitation assessment and in certain training monitoring settings, since physical exertion is not a prerequisite and the approach provides an acceptable VO_2_max estimation accuracy.

## Introduction

Maximal oxygen uptake (VO_2_max) is one of the parameters used to to assess the performance potential of endurance athletes^1–3^. VO_2_max is determined during incremental cardiopulmonary exercise testing (CPET) simultaneously with ventilatory thresholds allowing for the estimation of fixed and physiological training zones, respectively^4^. Additionally, VO_2_max determination is recommended during the training cycle to monitor the quality and to quantity the training progress^5^ or before the return to regular training regimens after a break^6^. Submaximal testing is sufficient for assessing functional limitations (equivalent to impairment observed in daily activities), verifying the outcome of interventions and evaluating the effects of pharmacological treatment in some patients^7^. However, VO_2_max remains the “gold standard” to assess functional capacity and cardiorespiratory responses to cardiovascular rehabilitation programs^8^. Direct VO_2_max determination requires laboratory conditions, appropriate equipment, sensor calibration and adherence to the guidelines for performing cardiorespiratory exercise testing^9^. Beyond the costs, laboratory CPET raises many organizational problems due to the standardization of experimental protocols and the need for an experienced and well trained staff. Further, in case of athletes, tests until exhaustion may interfere with their actual training plans, ambitions and may raise motivational issues during intense training periods. Since VO_2_max assessment requires high motivation and tolerance to exhaustive exercise, the probability of invalid tests and the risk of adverse events, especially in patient population, is increased.

Therefore, valid VO_2_max determination based on submaximal test can be a valuable alternative in clinical and sport applications. Lots of indirect methods have been developed, also taking into account the specificity of the sports discipline^3, 10^ or certain populations^2^. These indirect methods can be divided into two groups, i.e. those that require maximal effort (without monitoring VO_2_ kinetics) and those that rely on submaximal trials or resting parameters.

VO_2_max, or maximal oxygen consumption, represents the highest rate at which an individual's body can consume oxygen during intense exercise. To determine true VO_2_max, the traditional criterion has been introduced - the observation of a plateau in oxygen consumption despite an increase in exercise intensity. This plateau indicates that the individual has reached his physiological limit or maximal aerobic capacity. However, it's important to note that achieving this plateau can be subjective and challenging to define precisely. In clinical settings, when assessing patients with cardiovascular or pulmonary disease, it is often difficult to observe a clear plateau in oxygen consumption due to physiological limitations. Therefore, the term “VO_2_peak” is commonly used instead^11^. VO_2_peak represents the highest VO_2_ value achieved during an exercise test, even if a clear plateau is not observed. This term allows clinicians to express the patient's exercise capacity in a more practical and measurable way. On the other hand, in apparently healthy individuals without underlying cardiovascular or pulmonary conditions, it is more likely to observe a true VO_2_max response during exercise testing. Thus, the term “VO_2_max” is often used to describe their maximal physiological response^12^.

Incremental tests performed until the subject terminates the effort due to perceived exhaustion are the most common for the VO_2_max determination. Since both, a lack of experience in the experimental protocol and low motivation can distort its proper calculation: additional criteria were introduced for accuracy improvement and standardization of VO_2_max determination. Attaining of VO_2_ plateau was one of the main factors, but it met a lot of criticism^13, 14^. Further, the support by other markers was proposed and widely discussed as secondary criteria: respiratory exchange ratio, percentage age-adjusted estimates of HR, values of lactate concentration (usually > 7.9 mM^13^) and ratings of perceived exertion.

For the indirect exercise based methods, maximal power, covered distance, time to exhaustion or similar parameters are used to predict VO_2_max^15, 16^.

Similarly to the lab-based assessments, specific testing protocols and termination criteria are defined for indirect methods^2, 17^. Many aspects characterize the advantages of the field tests, but there are also limitations: poor control of the experimental conditions (which cause difficulties in repeatability of the test), bias due to the subjective assessments of physical activity or fitness in questionnaires^18^, sport specific approaches that may not only reflect the cardiorespiratory capacity but also technical factors, motivation or experience. Considering such limitations, one should expect an imperfection of indirect techniques, which is confirmed by validation procedures in relation to the measured VO_2_max^19, 20^. However, the usefulness of such field tests is wide: usually, many subjects can perform the experiment simultaneously, the cost of equipment is low and there is no need to use sophisticated calculation techniques. Further, many tests appear to be valid and safe for assessing group-level mean changes in VO_2_max. In result, the number of the indirect methods of VO_2_max estimation remains high in wide ranges of applications.

In general, the indirect methods do not consider the kinetics of physiological responses to progressive exercise in estimating VO_2_max, but focus on the parameters obtained at exercise cessation and/or on markers characterizing the subject (body mass, age, sex, etc.), only. These parameters are then used to feed regression models, that are constructed from population studies^15^. An optimal approach should guarantee not only the accurate estimation of VO_2_max but also a protocol that does not require complete exhaustion of the subject, i. e. submaximal testing. Such models are still rare because they require both monitoring of certain variables and dedicated computational tools^21^. However, they are attractive due to the elements of individualization, precision and submaximal effort^7^. Submaximal exercise testing often relies on the linear relationships between oxygen uptake and HR. Due to deflection at higher intensities such linearity may be not observed causing large error (about 15%) between extrapolated and true VO_2_max values^22^. Focusing on the submaximal tests requiring the cycle ergometer two are well known in the literature (and recommended for athletes): the Åstrand-Ryhming (A-R)^23^ and the YMCA protocol^2, 24^. Both tests are based on HR and thus has limited application to patients (especially these with pharmacological treatment affecting the HR)^22^. A-R is a 6 min test on a cycle ergometer with VO_2_max depending on both workload and HR. The modification contains an age correction factor. The second protocol is constructed from extrapolation of HR responses relative to power output increases. VO_2_max depends on age predicted maximal HR, from which maximal power output is found. Our proposal for VO_2_max prediction goes beyond these limitations. Here, the kinetics of VO_2_ and HR as a response to incremental workload are considered and modeled without the assumption of a linear relation between both variables. The maximal HR was also validated on the reference group resulting in the application of Whyte’s formula. Final extrapolation for VO_2_ is performed and the time series are obtained (not only final values, which is typical in other submaximal models). VO_2_max is found at the time point equivalent to the moment of attaining maximal HR in the model. This approach can be used for other populations with respect to successive steps described in the “Results” section. In the method not only the final values are determined, but the time evolution of both markers is presented in extrapolation.

## Methods

### Retrospective data and participants

Subjects: Data from incremental cycling tests recorded at the Institute of Sport Science of the University of Rostock (Germany) was selected for a retrospective study. In the analysis, time series from 17 healthy and young males (23.5 ± 2.0 years, BMI: 23.9 ± 3.2 kg/m^2^) were used. Participants considered for the study were classified as physically active (> 180 min of moderate to vigorous physical activity per week). All subjects were free of medication and resigned from intensive exercise and alcohol consumption for > 48 h prior to the test. Caffeine and nicotine were not allowed during the night and on the morning of the experiment. The volunteers performed an incremental cycling test on an SRP 3000 bicycle ergometer (Sportplus, Germany) until volitional exhaustion (VO_2_max test). Respiratory gas and volume analysis were carried out breath-by-beath using the MetaMax 3B system (Cortex Biophysics Inc., Germany). HR was monitored by the RS800 heart rate monitor (Polar Inc., Finland). The VO_2_max test included an adaptation (without cycling) lasting no more than 6 min, then 3 min warm-up at 50 W, incremental phase with step 25 W/min and at least 3 min cool-down at 50 W after effort termination. The study adhered to the ethical standards for research involving human subjects set in the declaration of Helsinki and was approved by the local ethics committee at the University of Rostock (A 2017-0034). All particpants gave their written informed consent.

### New experimental protocol for model validation and volunteers characterization

Ten subjects of both sexes (5/5) were recruited among physical education students of the University of Rostock. One female participant had to be excluded due to medications before the beginning of the study. Thus, time series from 9 subjects (five males: 24.6 ± 1.3 years, BMI 23.0 ± 1.1 kg/m^2^ and four females: 25.7 ± 3.1 years, BMI 23.4 ± 3.2 kg/m^2^) were included in the final validation analysis. The volunteers performed two incremental cycling tests on an SRM Ergometer (Schoberer Messtechnik GmbH, Germany) in the validation protocol: until volitional exhaustion (VO_2_max test) and until reaching 80% of their estimated HRmax, respectively, in a randomized order. Respiratory gas and volume analysis were carried out breath-by-beath using the MetaMax 3B system. HR was monitored by the RS800 heart rate monitor. The maximal protocol included 3 min of familarization, at least 5 min warm-up and a 5 min cool-down cycling phase, both at 50 W. The increments during the ramp phase were 25 W/min. Participants were verbally encouraged during the test until exhausion. The submaximal protocol was terminated during the ramp phase and afterwards 5 min recovery at 50 W workload started. The termination point (80% of estimated HRmax) was estimated from retrospective analysis (details are given in Sect. “A method for determination of the termination point in a step incremental tests”).

### Stages of model construction

#### Estimation of HRmax and VO_2_max from resting parameters

The proposed method of VO_2_max determination relies on the HRmax estimation. For comparison purposes, the estimation of the VO_2_max was also required. Note, that in both markers, only equations which consider resting parameters were taken into consideration. For clarification, the concepts used in the text: estimation is dedicated to determination of maximal parameters from equations using resting markers, while prediction is obtained from differential models.

We verified the agreement of the retrospective data with the results estimated from linear equations with resting parameters. For the analysis, five methods for HRmax estimation were selected^25–29^ and three for VO_2_max^30–32^. The quality of agreement between estimated and experimental values was assessed by the mean δ̅ calculated from all relative errors δ (Eq. 1):

1 $$\delta = \frac{{\left| {X_{experimental} - X_{predicted} } \right|}}{{X_{experimental} }} \cdot 100\% .$$

Finally, we selected the model introduced by Whyte et al.^25^ for HRmax estimation (δ̅ = 5.73%):

2 $$HRmax_{{}} = \left\{ {\begin{array}{*{20}l} {202 - 0.55 \cdot age, \; for\; males} \\ {216 - 1.09 \cdot age, \;for \;females.} \\ \end{array} } \right.$$

In case of VO_2_max estimation using our retrospective data, we found the smallest mean relative error δ (8.37%) for the Jurca’s equation:

3 $$VO_{2} {\text{max}}\left[ {\frac{ml}{{kg \cdot min}}} \right] = 3.5 \cdot \left[ {18.07 + 2.77 \cdot sex - 0.1 \cdot age - 0.17 \cdot BMI - 0.03 \cdot RHR + PAS} \right],$$

Where, sex = 1 for males, and 0 for females, RHR is a resting HR, and PAS reflects the usual pattern of daily physical activities. The latter one, was indexes for our subjects at the highest level, while they indicated at least 3 h of intense physical activity per the week.

#### On the mathematical model for HR and VO_2_ kinetics

In^21^ a model in form of first-order differential equation was developed for the representation of the VO_2_ and HR responses to the workload *P(t)* set on cycle ergometer:

4 $$\frac{dX}{{dt}} + \frac{{X\left( t \right) - X_{0\_val} }}{\tau } = \frac{K}{\tau } \cdot P\left( t \right)$$

P(t) is interpreted as excitation variable and can be also considered for treadmill tests^21^ with relation to speed. X(t) represents the indices of HR or VO_2_. Three parameters must be determined from experimental data for further prediction: (1) X_0_val_, which is an equilibrium value (val) of HR or VO_2_, observed at resting conditions—*P(t)* = *0*. (2) K—represents the gain^21^ related to the magnitude of increase the HR (or VO_2_) in the response to increase magnitude of the power P. Precisely, using example, when K_HR_ is 2 beats/min/W, then 25 W increase in power will result in an increase of 50 heartbeats per minute (bpm). The 50 W increase in power will double the increase of the HR, leading to a change equal to 100 bpm. The last parameter is a time decay *τ*. It is a period of time, in which the variable reaches the 63% of the absolute change under a constant work increase. The prediction procedure of these three basic model parameters was described in detail by Mongin et al.^21^. It should be underlined that the authors presented the dedicated numerical algorithm with an open-source package doremi (R software—^33^), which was used in our analysis.

In^21^, Eq. (4) was successfully validated with experimental data and presented as a promising tool for the extrapolation of VO_2_ and HR indices from submaximal test protocols, which were constructed from maximal trials. Our study is an extension of this analysis. We proposed and discussed the determination of time point, when the incremental test should be stopped allowing for most optimal prediction accuracy of VO_2_max in accordance with maximal HR. In our study, model parameters determination relies on VO_2_ and HR kinetics recorded during a submaximal test. The approach represents true physiological responses for incremental submaximal testing that may differ from artificially composed truncated tests especially with regard to the recovery dynamics. Similar protocols (not maximal) are proposed for further applications in both clinical and sport practice.

#### A method for determination of the termination point in a step incremental tests

Starting with the retrospective data, the following procedure was developed. Using the idea presented in the paper^21^. The retrospective time series recorded during the incremental phase were truncated. For test purposes, the time moment of cutting off the data was selected in relation to the experimental HRmax. At the beginning, the VO_2_ recording was truncated, when the HR reached accordingly: 71%, 72%,…, 94%, and 95% of HRmax. The corresponding value of VO_2_ from the recovery phase was detected and the time series below were treated as recovery for truncated data. A similar procedure was done for HR signals. Each truncated dataset was studied separately. Note that originally Mongin et al.^21^ applied the signal cutting at the moment, that fell between two ventilatory thresholds in order to examine the change of performance of the young athletes. Here, we proposed a dedicated approach for time detection, which relies on the validation of VO_2_max prediction.

In our study, truncated time series were considered as the submaximal effort with recovery and warm-up. Using the differential model (Eq. 4) with three estimated parameters (*K, τ, X*_*0*_) the extrapolation of HR data was performed until it reached the maximal estimated value. The time step in the process of extrapolation was 1 s and the accuracy of the HR was set to 0.1 bpm. The moment in time for attaining the VO2max in the extrapolation procedure was equivalent for the timewhen estimated HRmax was found in the extrapolated data.

**Figure 1:**
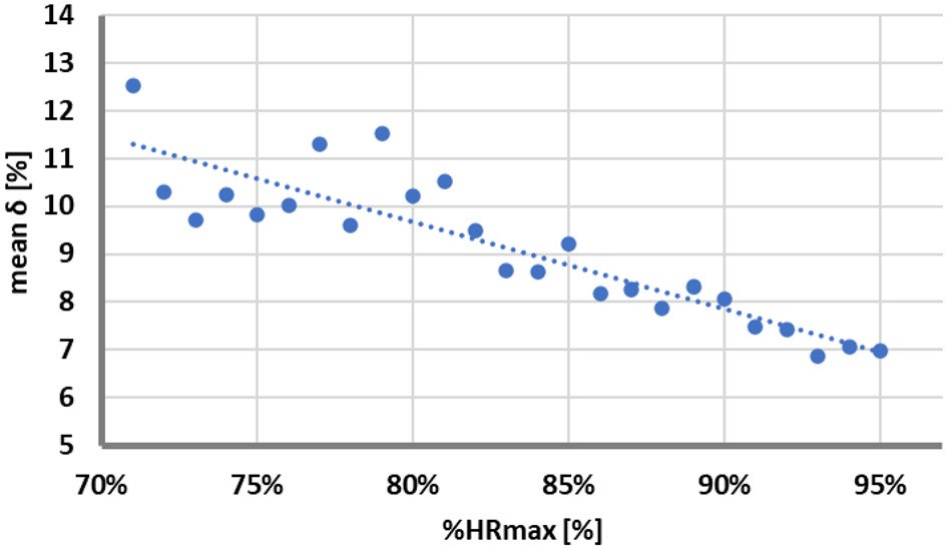
The plot representing the relation between mean error δ (from retrospective data) and the moment of experimental data truncation in construction recordings representing the submaximal test. Decreasing linear fit is observed (coefficient of determination R^2^ = 0.78), indicating increasing accuracy of prediction with increasing data length used for extrapolation.

The final step was dedicated to the assement of the correspondence between experimental VO_2_max and the value determined by the extrapolation in our proposal. Note, that the analysis was done on the retrospective data. The comparison was again quantified by relative error (Eq. 1). As expected, the shorter data representing submaximal test, the larger difference between experimental and VO_2_max predicted (from extrapolation)—see Fig. 1. The correlation between mean relative error and %HRmax is high (*ρ* = *− 0.89*, *p-value* < *0.001*) showing impact of the time duration of the submaximal test on the accuracy of the VO_2_max prediction.

This numerical experiment was necessary to find an acceptable moment of termination of the incremental tests in the applied setting. This decision was a compromise between the observed magnitudes of fluctuations of the mean relative error for short submaximal datasets (Fig. 1) and the level of exertion/fatigue. The shorter the test duration and thus the load, the lower the level of exertion/ fatigue and the risk for adverse event in (sub)clinical populations as well as patient compliance. Similarly, submaximal ramp test protocols are well tolerated in athletic populations and may therefore allow repeated testing schemes within short time frames. On the other hand, short data length reduces the accuracy of the VO_2_max prediction. The intraindividual day-to-day variation in determination VO_2_max ranges between 4 and 6% in healthy subjects with no known pathology or impairment^7^. In case of patients, this variation is even higher and reaches 10%^34, 35^. Additionally not only difficulties in data repetition should be considered but also the measurement error of the ergospirometry device itself (usually around 1%). Considering all implications, we proposed to terminate the submaximal incremental test, when HR of the subject is reaching 80% of the estimated HRmax from linear resting model (Eq. 2).

## Results

### Submaximal model for oxygen uptake prediction (SMO)—protocol

SMO uses a mathematical equation (Eq. 4) for the determination of the dynamics for HR(t)—HR as a function of time and for VO_2_(t) in response to the increasing workload on the cycle ergometer. Additionally, the workload *P(t)* settings for the incremental cycle test are necessary as input data. The proposed SMO method should allow for improved accuracy in the prediction of VO_2_max than methods relying on resting parameters, only. The final experimental protocol for the developed SMO is following:

1. Estimation of HRmax of the examined subject using a published regression formula (Eq. 2).
2. Performing the incremental cycle test (containing baseline, warm-up, incremental phase and recovery). The incremental phase is stopped, when the subject reaches the 80% of HRmax estimated in point 1. The following data are recorded during experiment: oxygen uptake (sampling rate 1 Hz), HR. The raw HR(t) and VO_2_(t) signals where preprocessed by moving average filter in 15 measurement points window.
  Note that we studied young, active subjects without any known diseases, completing a relatively sharp, but established, incremental test protocol. In case of adapted workload protocols in clinical populations, we highly recommend repeating the procedure described in Sect. “A method for determination of the termination point in a step incremental tests” on retrospective data. The moment for submaximal termination of the test to approach sufficient accuracy as well as to consider patient safety aspects may differ.
3. Determination of the three characteristic parameters (*τ*_*HR*_*, K*_*HR*_*, X*_*0_HR*_) of the model (Eq. 4) for the HR(t) signal recorded in the submaximal test under 2. For this purpose, we used the numerical algorithm given in open-source package *doremi*^33^. The package was also applied in the next steps (4–6) of the procedure.
4. Extrapolation of HR(t) dynamics by the mathematical model and parameters determined under 3. Note that in extrapolation, the same step increments for load must be applied. The extrapolation is performed until the HR reaches the HRmax value determined under 1. The moment of time of this event is denoted as *t*_*HRmax_model*_.
5. Repetition of the procedure described under 3 for the VO_2_ signal. In result, three characteristic parameters (*τ*_*VO2*_*, K*_*VO2*_*, X*_*0_VO2*_) are obtained.
6. Extrapolation of VO_2_(t) dynamics by the mathematical model and parameters determined under 5. The values for *P(t)* must be the same as used in modeling the HR kinetics (see 4.). The extrapolation algorithm is stopped at *t*_*HRmax_model*_. The resultant value is found to be VO_2_max, which corresponds to the predicted maximal HR. The recovery phase also may be extrapolated by introducing the workload during the recovery phase (for example as in our study 50 W) in the model.

### Comparison of the predicted maximal oxygen uptake with the experimental data

**Figure 2:**
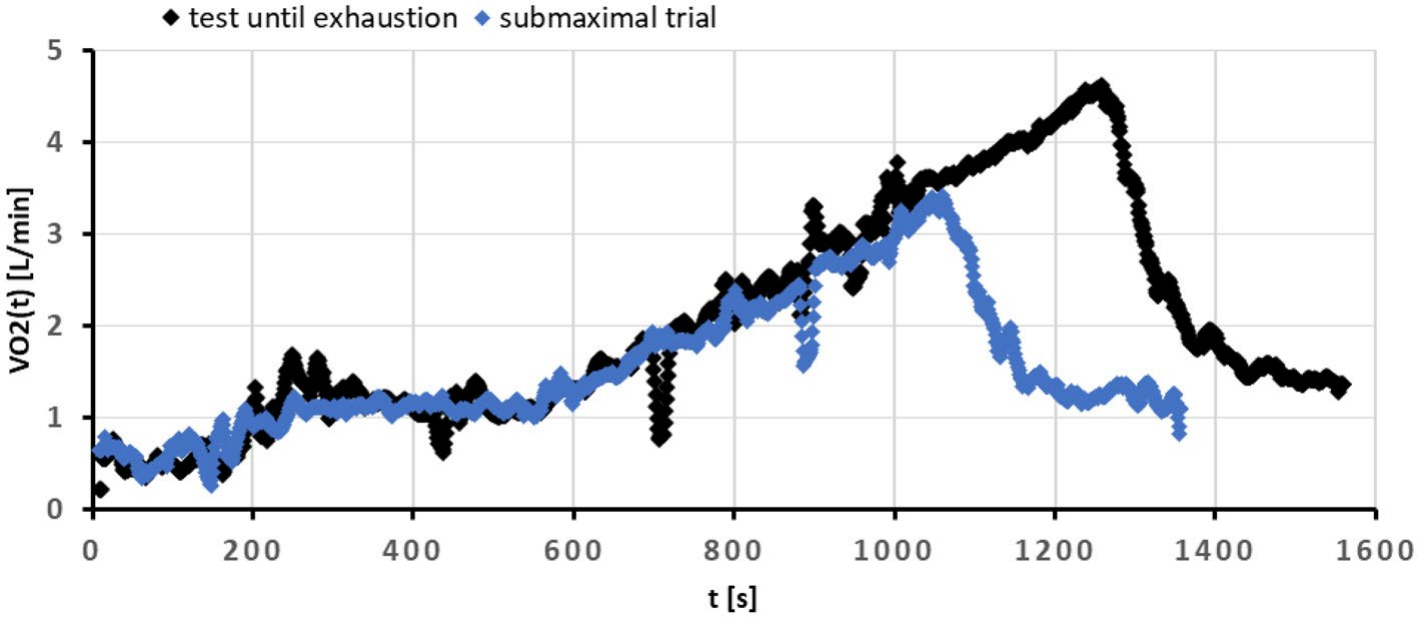
Typical recording from incremental tests (data after preprocessing, see details in Sect. “Submaximal model for oxygen uptake prediction (SMO)—protocol”): black points represent the test until exhaustion, blue the submaximal trial. Note that the recovery phase differs between trials (slower decrease is observed for the submaximal test).

Two trials (details in Sect. “New experiment protocol for model validation and volunteers characterization”) were performed by the subjects of the second experimental group to test the accuracy of the VO_2_max from the SMO model. The typical preprocessed recordings representing both, the incremental test until volitional exhaustion and the submaximal test, terminated at 80% of estimated HRmax, are shown in Fig. 2.

Predicted VO_2_max values determined by our approach were compared with experimental data. In Table 1, we presented three markers (VO_2_max values determined from experiment, from SMO prediction and from Jurca’s estimation) and their relative errors. The mean relative error δ  ± SD is found to be equal 5.56 ± 3.92% for SMO. The prediction of VO_2_max is more acurate than the estimation given by Jurca’s model (Eq. 3), for which mean relative error equals 10.1% (SD = 5.7%).

**Table 1:** Comparison of methods: prediction based on the submaximal model (SMO) and estimation using resting parameters (Jurca's model) with the experimental results for VO_2_max obtained during incremental step test.

Subject	VO_2_max experimental $\left( {\frac{L}{{{\text{min}}}}} \right)$	VO_2_max SMO $\left( {\frac{L}{{{\text{min}}}}} \right)$	${\updelta }_{SMO}$ (%)	VO_2_max Jurca $\left( {\frac{L}{{{\text{min}}}}} \right)$	${\updelta }_{Jurca}$ (%)
S1	3.54	3.47	1.9	3.09	12.7
S2	4.57	4.80	5.1	4.20	8.1
S3	4.34	3.76	13.4	4.16	4.1
S4	3.35	3.55	6.1	3.41	1.9
S5	4.58	4.98	8.7	4.02	12.3
S6	3.29	3.23	1.9	2.71	17.8
S7	4.02	4.20	4.5	4.36	8.5
S8	3.68	3.64	1.2	3.00	18.5
S9	3.97	4.26	7.4	3.68	7.5

## Discussion

The main goal of the study was to develop the protocol for submaximal test in order to predict VO_2_max. Using recently studied model for HR and VO_2_ (Eq. 4,^21^), we described in detail the methodology for the justification of the termination point for submaximal tests. The procedure requires submaximal tests with oxygen uptake monitoring. The need for metabolic testing equipment may be viewed as a limiting factor of the proposed method. However, decision making with respect to training and rehabilitation may benefit from the evaluation of the kinetics of the cardiorespiratory response. The model is currently applicable to a standard incremental test scheme on a cycle ergometer. Cycling is considered for rehabilitation mode^36^ in cardiac patients but future extension of this prediction to treadmill exercise is recommended. We believe that similar models have the potential to test symptomatic patients during and after rehabilitation, for whom maximal effort is not always recommended and feasable. Current opinions^7, 23^ indicate that such submaximal testing (also on cycle ergometer) are better applicable in several patient populations, including subjects with impaired balance, overweight subjects and patients exhibiting decrease exercise tolerance due to other risk factors and conditions. Even patients without diagnosed cardiac disorders (especially older one) may exhibit arrhythmias during tests (in response to effort). Using submaximal test instead of maximal one represents an approach, in which the safety and comfort of patient is principal^7^. Submaximal tests are in line with recent recommendations of American Heart Association related to assessing cardiorespiratory fitness in the practice^37^, where (point 1) “clinicians may consider the use of submaximal exercise tests or field tests as alternatives, because these involve individual-specific exercise responses”. What is more, VO_2_ kinetics in submaximal tests better reflects daily functional capacity of heart failure patients than parameters measured at maximal exertion, and may therefore better predict effects of medical interventions (example in resynchronization therapy—^38^). In case of healthy subjects, the data determined from the differential model (Eq. 4) with significant correlation^21^ reproduce the ventilatory markers of physical performance and its changes e. g. during (de)conditioning in athletes.

On the other hand methods predicting VO_2_max from submaximal tests have certain limitations in practical applications due to physiological changes in the kinetics of VO_2_ and HR: experimental VO_2_(t) can be characterized by a plateau during very high-intensity exercise^39^ and sometimes a deflection in the HR(t) after the second ventilation threshold can be observed^21, 40^. Such form of dynamics is difficult to predict in early terminated experiments (here 80% of HRmax). The next relevant aspect of modeling and testing the accuracy of the method is VO_2_max determination itself. In recommendations one may find many different criteria^13, 14, 41^ to make sure that the measured VO_2_peak value equals the true VO_2_max. The differential equation used in the SMO method origins from steady state model, where slow component phase is considered^42^ with relation to VO_2_max. Simultaneously, we obtained good agreement with VO_2_max determined in maximal tests (mean error 5.56%), which confirms the quality of the SMO for VO_2_max prediction in our healthy sample. However, in case of other groups, the method must be verified, especially when disorders (in metabolism, oxygen transport etc.) related to oxygen kinetics may be expected^43^.

A brief summary of reliability of popular submaximal tests (not limited to cycling) are presented in^7^. Considering the A-R protocol (see “Introduction”) a lot of validation studies were performed. For example, in a group of men (age 30–66 years) results obtained from test underpredicted VO_2_max by 21%. I case of women (age: 19–70 years), A-R method overestimated VO_2_max up to 21%. Values obtained from the revised A-R method (revised nomogram) in a group of women (19–47 years) indicated overestimation of VO_2_max by 18.5% [^7^ and references therein]. Results of validation for YMCA protocol revealed an overestimation for both sexes^44, 45^, while in^45^ percentages are the lowest: 5.4% for men and 11.8% for women, respectively. Concluding, the prediction accuracy of our submaximal approach (with mean error 5.56%) should suffice to detect typical improvements in VO_2_max within cardiac rehabilitation programs (around 5–10% on average)^7, 37^ even if VO_2_max is assessed with different protocols. Applying the same (submaximal) protocol for within-subject testing should provide a better sensitivity and might improve the detection of meaningful changes in VO_2_max in rehabilitation settings. Additionally, the changes in VO_2_ kinetics (at lower workloads) may be observed even in daily variability, for example due to familiarization to the protocol and a second trial might improve accuracy. However, until now, no data on test–retest reliability can be provided for our submaximal protocol. In the future, criteria such as ventilatory thresholds, heart rate variability thresholds, rating of perceived exertion, the respiratory exchange ratio, HR recovery kinetics or similar submaximal indices should be considered as potential covariates in the model to evaluate and correct for the “true” individual submaximal level and therefore improve the accuracy and applicability of the model on individual- and group-level.

## Data Availability

The datasets generated during and/or analysed during the current study are available from the corresponding author on reasonable request.
